# Adaptive Finite-Time-Based Neural Optimal Control of Time-Delayed Wheeled Mobile Robotics Systems

**DOI:** 10.3390/s24175462

**Published:** 2024-08-23

**Authors:** Shu Li, Tao Ren, Liang Ding, Lei Liu

**Affiliations:** 1 The Key Laboratory of Intelligent Control Theory and Application of Liaoning Provincial, Liaoning University of Technology, Jinzhou 121001, China; rentaolab2022@163.com (T.R.); liuleill@live.cn (L.L.); 2 The State Key Laboratory of Robotics and System, Harbin Institute of Technology, Harbin 150001, China; liangding@hit.edu.cn

**Keywords:** approximate dynamic programming, wheeled mobile robotics, tracking control, state time delay, finite time

## Abstract

For nonlinear systems with uncertain state time delays, an adaptive neural optimal tracking control method based on finite time is designed. With the help of the appropriate LKFs, the time-delay problem is handled. A novel nonquadratic Hamilton–Jacobi–Bellman (HJB) function is defined, where finite time is selected as the upper limit of integration. This function contains information on the state time delay, while also maintaining the basic information. To meet specific requirements, the integral reinforcement learning method is employed to solve the ideal HJB function. Then, a tracking controller is designed to ensure finite-time convergence and optimization of the controlled system. This involves the evaluation and execution of gradient descent updates of neural network weights based on a reinforcement learning architecture. The semi-global practical finite-time stability of the controlled system and the finite-time convergence of the tracking error are guaranteed.

## 1. Introduction

Adaptive intelligent control algorithms have developed rapidly with the advancement of intelligent approximation technology, especially in neural networks (NN) and fuzzy logic systems (FLS), and have achieved a series of excellent research results [1,2,3,4,5,6,7,8,9]. This has also significantly motivated many scholars to explore the adaptive control algorithm, laying a solid foundation for using the corresponding control theory algorithm in the field of practical engineering applications.

Considering that control and decision-making problems are essentially optimization problems, and optimal control plays a key role in engineering applications, the research on intelligent majorization control algorithms in this paper has a certain role in promoting practical engineering applications. In view of the importance of optimal control, many scholars have conducted extensive research on optimal control algorithms and have obtained certain achievements, mainly including two optimization methods [10,11], adaptive dynamic programming (ADP) methods [12] and reinforcement learning (RL) methods [13].

The ADP approach could realize the online approximation of the optimal target by the recursive numerical method without relying on the control algorithm of the model [14,15,16,17,18,19]. Using NNs, the performance function, designed control laws, and the uncertain part of the nonlinear system could be approximated, which helps solve the HJB function; then, the optimal stability is guaranteed. Similar to the learning mechanism of mammals, the reinforcement learning mechanism aims to regulate both the critic and action adaptive laws in order to control the long-term interaction cost of the environment. The action NNs could modify the action laws, while the critic NNs reduce the virtual energy of the long-term storage function. Thanks to the interoperability of the operating mechanism, refs. [20,21,22,23,24] have made outstanding contributions to online optimization control and model-free optimization control.

Although previous ADP-based methods perform well for non-linear systems without time delays, achieving the ideal control effect on time-delayed non-linear systems is often challenging. Therefore, research on this topic has generated interest among experts and scholars and has achieved preliminary results. However, the time delay in the form of nonlinear interference is a major obstacle to applications of control theory algorithms. Some scholars have paid attention to this and have achieved certain results. Regarding existing methods, there are two main forms of system delay: state and input [25].

State time delays are mainly found in intricate engineering systems, for example, wheeled mobile robot (WMR) systems and chemical engineering, which are hysteresis induced by internal propagation of signals during system motion. With assistance from the Lyapunov–Krasovskii functional (LKF) [25,26,27], the influence caused by the state time delay is overcome, and superior control algorithms are designed.

Due to the contradiction between the convergence characteristics of existing optimization algorithms in infinite time and the fast convergence requirements of actual engineering systems, it has greatly inhibited the practical application and promotion of intelligent optimization algorithms. Therefore, in recent years, some scholar award research has focused on the study of convergence speed and convergence domain equilibrium. The existing breakthrough theoretical research results on infinite convergence algorithms [28,29,30,31,32,33] have promoted the research process of finite-time convergence control algorithms to a certain extent and also reflect the necessity of studying the algorithm from the side. At the same time, they also point out the key and difficult issues faced by finite-time convergence control research.

To meet the finite-time or finite-horizon domain convergence characteristics of actual engineering requirements, some scholars have begun relevant research. For nonlinear discrete systems, researchers use the ADP-based approach to solve the finite-time domain convergence problem [34,35], which greatly stimulates the authors’ research passion for finite-time convergence optimization control algorithms.

Different from finite-horizon convergence, besides guaranteeing the time domain of system convergence, finite-time convergence also increases the speed and accuracy of system convergence. However, the existing research is not perfect and is still in its infancy, but some studies with excellent performance have been obtained [36,37,38,39,40,41,42,43,44,45]. Up to now, the finite-time optimization algorithm, considering both convergence speed and convergence precision as well as considering energy consumption, is basically absent. Therefore, based on the previous research, this paper not only considers the state delay, but also considers the input delay, and uses the ADP method to effectively resolve the finite-time optimal tracking control problem of the controlled target.

An adaptive finite-time online optimal tracking control method based on neural networks is designed for uncertain nonlinear systems with state time delays. Firstly, the initial nonlinear system is extended to an augmentation system, which contains tracking error and target expectation information, and a novel discounted performance function is presented. Secondly, a Hamiltonian function is constructed, and the appropriate LKFs are used to resolve the problem of state delay. Then, for the solution of the ideal HJB function, this paper introduces the method of integral reinforcement learning (IRL). Finally, by designing the optimal control strategy and optimizing the control adaptive law, the semi-global practical finite-time stability (SGPFS) lemma, not only is the influence of time delays eliminated, but the stability of uncertain nonlinear systems is guaranteed. The main innovative work includes:

(1) The time-delay effect is incorporated into the strategy design process to address the finite-time convergence issues.
(2) The problem caused by the state time delay is solved simultaneously in the optimal control process.
(3) The optimal control policy guarantees that the target control system achieves optimal control within a finite time.

## 2. System Description and Preliminaries

Considering the state time-delayed nonlinear system as

(1) $$\dot{\beta }\left(t\right)=p\left(t\right)\beta \left(t\right)+h\left(\beta \left(t-t_{1}\right)\right)+g\left(t\right)u\left(t\right)+\omega \left(t\right)$$

where the delayed dynamics $h\left(\beta \left(t-t_{1}\right)\right)$ is one known function vector with an unknown time delay $t_{1}$. For the sake of simplicity in subsequent expressions, except for the hysteresis term $\beta \left(t-t_{1}\right)$, t and other variables are omitted. $g\left(t\right)$ denotes the input function, $p\left(t\right)$ denotes the state function, $u\left(t\right)$ denotes the system control input, and $\omega \left(t\right)$ denotes the external perturbation function.

Considering the state and the input time delays in system (1), the appropriate LKF is introduced to deal with the state time-delay problem, respectively. And according to Remark 1 in [26], only when the delayed dynamics $\alpha \left(t\right)$ are known, one can obtain $h\left(\beta_{1}\right)-h\left(\beta_{2}\right)={\left.\partial h\left(\beta \right)/\partial \beta \right|}_{\beta =\beta^{\kappa }}\left(\beta_{1}-\beta_{2}\right)$ with $\beta^{\kappa }=\kappa \beta_{1}+\left(1-\kappa \right)\beta_{2}$ and 0<κ<1.

The following scientific assumptions are made, and corresponding lemmas are given to ensure that the subsequent design process achieves the expected control objectives.

**Assumption** **1.** *Both function* $p\left(t\right)$ *and* $g\left(t\right)$ *are continuously differentiable. For the time-delay function* $p\left(\cdot \right)$*, its Jacobi matrix* $\partial p\left(\beta \right)/\partial \beta$ *satisfies the Lipchitz condition* $\left\|\partial p\left(\beta \right)/\partial \beta \right\|\leq \eta$ *with* η≥0.

**Assumption** **2.** *The boundedness of the unknown input transfer function* $g\left(t\right)$ *can be obtained as* $\underline{g}<g\leq \bar{g}$*. Similarly,* $\sigma_{\min}\leq \left\|\sigma \left(\cdot \right)\right\|\leq \sigma_{\max}$*;* $\varphi_{\min}\leq \left\|\varphi \left(\cdot \right)\right\|\leq \varphi_{\max}$ *can be used to present the boundedness of the activation functions in hidden layers of NNs* $\varphi \left(\cdot \right)$ *and the functional approximation error* $\sigma \left(\cdot \right)$.

**Lemma** **1** **([44]).** *For any states* $y_{i}\in R$*,* i=1,2,…,m *, if the positive constant satisfies* 0<q<1 *, we have*

(2) $${\left(\sum_{i=1}^{m}\left|y_{i}\right|\right)}^{q}\leq {\sum_{i=1}^{m}\left|y_{i}\right|}^{q}\leq m^{1-q}{\left(\sum_{i=1}^{m}\left|y_{i}\right|\right)}^{q}$$

**Lemma** **2** **([39]).** *For the nonlinear system $\dot{x}=f\left(x\right)$, if (3) holds,*

(3) $$\dot{L}\left(x\right)\leq -\iota L^{b}\left(x\right)+\sigma ,t\geq 0$$

*where $L\left(x\right)$ is a smooth positive definite function, ι>0, 0<b<1, σ>0, one can further obtain that the nonlinear system $\dot{x}=f\left(x\right)$ is SGPFS.*

In this paper, by designing an adaptive NN-based optimal controller $u\left(t\right)$ such that $\beta \left(t\right)$, the output of the system could track $\beta_{d}\left(k\right)$ well in a finite time. The two main types of neural networks used in this paper include critic neural networks and action neural networks. The critic neural network is used for the estimation of the long-term utility function, while the action neural network is used to ensure the stability of the system and the solution of the optimal control inputs of the system.

## 3. Controller Design and Stability Analysis

**Figure 1:**
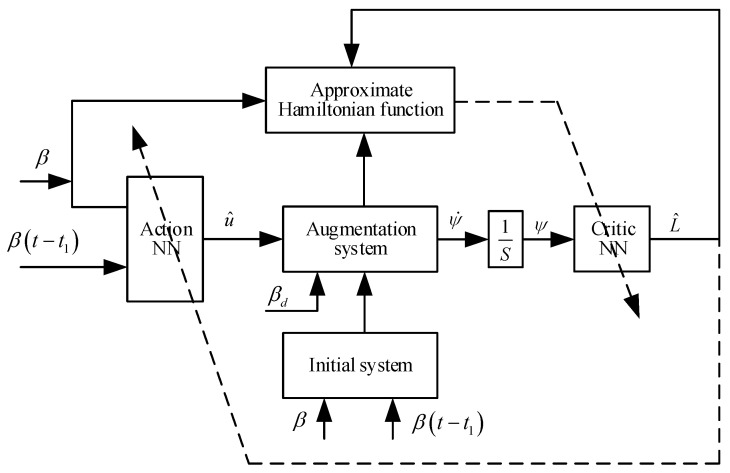
Finite time convergence adaptive optimal tracking control algorithm structure drawing.

This section is divided into subheadings, which provide a concise and precise description of the experimental results and their interpretation, as well as the experimental conclusions that can be drawn. Depicted in Figure 1, in this section, we design an optimal controller, which ensures the optimal control of the system and converges within a finite time. By transforming the initial system into an augmented system, which tracks errors and targets expected information, a novel discounted performance function is presented. Furthermore, a Hamiltonian function is constructed, and the time-delay problem will be solved by using the appropriate LKFs. Then, by introducing the IRL method to the Hamiltonian function, a finite-time optimal tracking controller based on neural networks is designed. Finally, the adaptive law of the appropriate evaluator and the adaptive law of the action NN are designed; the target system’s SGPFS can be ensured.

### 3.1. System Transformation

Considering the nonstrict nonlinear system (1), we developed a controller using a neural network to enable the system to follow the desired trajectory. Firstly, the tracking error system can be design as

(4) $$z\left(t\right)=\beta \left(t\right)-\beta_{d}\left(t\right)$$

Then, find the (4) derivative, and we can obtain

(5) $$\dot{z}=p\left(t\right)\beta \left(t\right)+h\left(\beta \left(t-t_{1}\right)\right)+g\left(t\right)u\left(t\right)+\omega \left(t\right)\left(t\right)-{\dot{\beta }}_{d}$$

**Assumption** **3.** *The target-given trajectory* $\beta_{d}$ *with the initial state as* $\beta_{d}\left(0\right)=0$ *is bounded, and* ${\dot{\beta }}_{d}\left(t\right)$ *can be rewritten into the form of (6) by a command generator function that satisfies the Lipschitz continuity property.*

(6) $${\dot{\beta }}_{d}\left(t\right)=l\left(\beta_{d}\left(t\right)\right)$$

The algorithm is expected to adopt a new type of discounted performance function, which includes both tracking error terms and expected trajectories and time-delay terms. Therefore, we constructed the following widening system.

(7) $$\dot{\psi }\left(t\right)=F\left(\psi \left(t\right)\right)+H\left(\psi \left(t-t_{1}\right)\right)+G\left(t\right)u\left(t\right)+W\left(t\right)$$

where $\psi \left(t\right)=\left[z\left(t\right),\beta_{d}\left(t\right)\right]$, $F\left(\psi \left(t\right)\right)=\left[\begin{array}{c} F_{1}\left(t\right)\left(z+\beta_{d}\right)-l\left(\beta_{d}\right)\\ l\left(\beta_{d}\right) \end{array}\right]$, $H\left(\psi \left(t-t_{1}\right)\right)=\left[\begin{array}{c} H_{1}\left(z\left(t-t_{1}\right)+\beta_{d}\left(t-t_{1}\right)\right)\\ 0 \end{array}\right]$, $G\left(t\right)=\left[\begin{array}{c} G_{1}\left(t\right)\\ 0 \end{array}\right]$, $W\left(t\right)=\left[\begin{array}{c} D_{1}\left(t\right)\\ 0 \end{array}\right]$.

Furthermore, the novel discounted performance function is

(8) $$L_{1}\left(t\right)=\int_{t}^{t+t_{0}}e^{-\chi \left(\tau -t\right)}\left(\Gamma^{T}Q\Gamma +U\left(u\right)\right)d\tau$$

where $\Gamma ={\left[\psi \left(t\right),\psi \left(t-t_{1}\right)\right]}^{T}$, χ is the discount factor, with χ>0 is a constant, and $Q=\left[\begin{array}{cc} Q_{1} & 0\\ 0 & Q_{2} \end{array}\right]$, where $Q_{i}$ is a matrix that is positive definite, and $t_{1}$ satisfies $t\geq t_{1}$, and the semi-global uniform convergence in (7) can be ensured with $t\geq t_{1}$.

Based on [46,47,48,49,50,51,52], and taking the input constraints into consideration, the nonquadratic functional is proposed as

(9) $$U\left(u\right)=2\int_{0}^{u}{\left(\varepsilon {\;\tanh}^{-1}\left(v/\varepsilon \right)\right)}^{T}Rd_{v}$$

where ε is the saturation input, and $R=diag\left(r_{1},r_{2}\right)$, $U\left(u\right)$ is a non-quadratic matrix.

### 3.2. Virtual Control

In this part, based on the Hamiltonian function, which is established based on the discounted performance function, the virtual optimal controller $u^{*}\left(t\right)$ will be designed.

To obtain the tracking Bellman equation, we used the Leibniz rule and (9) to obtain

(10) $${\dot{L}}_{1}=\chi L_{1}-\left(1-e^{-\chi t_{0}}\right)\left[\Gamma^{T}Q\Gamma -2\int_{0}^{u}{\left(\varepsilon_{0}{\;\tanh}^{-1}\left({v/\varepsilon }_{0}\right)\right)}^{T}Rd_{v}\right]$$

Then, we moved the right-hand side of Equation (10) to the left-hand side of the equation and substituted it into Equation (8) to finally obtain Equation (11)

(11) $$V=\left(1-e^{-\chi t_{0}}\right)\left[\Gamma^{T}Q\Gamma +2\int_{0}^{u}{\left(\varepsilon {\;\tanh}^{-1}\left(v/\varepsilon \right)\right)}^{T}Rd_{v}\right]\;-\chi L_{1}+\frac{\partial L_{1}}{\partial \Gamma }\left(F\left(\psi \right)+Gu+H\left(\psi \left(t-t_{1}\right)\right)+W\right)=0$$

In addition, we designed the optimal cost function in (12) from

(12) $$L_{1}^{*}\left(t\right)=\underset{u}{\min}\int_{t}^{t+t_{0}}e^{-\chi \left(\tau -t\right)}\left(\Gamma^{T}Q\Gamma +U\left(u\right)\right)d\tau$$

the following conditions should be guaranteed

(13) $$V^{*}=\left(1-e^{-\chi t_{0}}\right)\left[\Gamma^{T}Q\Gamma +2\int_{0}^{u^{*}}{\left(\varepsilon {\;\tanh}^{-1}\left(v/\varepsilon \right)\right)}^{T}Rd_{v}\right]\;-\chi L_{1}+\frac{\partial L_{1}^{*}}{\partial \Gamma }\left(F\left(\psi \right)+Gu^{*}+H\left(\psi \left(t-t_{1}\right)\right)+W\right)=0.$$

Based on (11) and [53], and the finite-time convergence theory [34], the optimal control input defined as

(14) $$u=-\varepsilon \;\tanh\left(l_{1}\psi^{2b-1}+\frac{\varepsilon r^{-1}G^{T}}{2\left(1-e^{-\chi t_{0}}\right)}\frac{\partial L_{1}}{\partial \Psi }\right).$$

According to (13) and [44,53], the ideal optimal control input is abbreviated as

(15) $$u^{*}=-\varepsilon \;\tanh\left(l_{1}\psi^{2b-1}+\Xi \frac{\partial L_{1}^{*}}{\partial \Psi }\right)$$

where $l_{1}=\frac{R^{-1}}{2\varepsilon }$ is a constant greater than zero and $\Xi =\frac{r^{-1}G^{T}}{2\varepsilon \left(1-e^{-\chi t_{0}}\right)}$.

Then, together with (14), (9) can be written as the following form

(16) $$U\left(u^{*}\right)=2\varepsilon^{2}{\left(l_{1}\psi^{2b-1}+\Xi \frac{\partial L_{1}^{*}}{\partial \Gamma }\right)}^{T}\;\tanh\left(l_{1}\psi^{2b-1}+\Xi \frac{\partial L_{1}^{*}}{\partial \Gamma }\right)\;+\varepsilon^{2}R_{0}\ln\left(E_{0}-\tanh^{2}\left(l_{1}\psi^{2b-1}+\Xi \frac{\partial L_{1}^{*}}{\partial \Gamma }\right)\right)$$

where $R_{0}=\left[R_{1},\ldots ,R_{m}\right]$ and $E_{0}=\underset{m}{\underbrace{\left[1,\ldots ,1\right]}}$.

The Hamiltonian function can be written as the following form

(17) $$V^{*}=-\chi L_{1}^{*}+\left(1-e^{-\chi t_{0}}\right)\psi^{T}Q\psi +2\varepsilon^{2}\left(1-e^{-\chi t_{0}}\right)l_{1}\psi^{2b-1}\;\tanh\left(l_{1}\psi^{2b-1}+\Xi \frac{\partial L_{1}^{*}}{\partial \Gamma }\right)\;+\frac{\partial L_{1}^{*}}{\partial \Gamma }\left(F\left(\psi \right)+H\left(\psi \left(t-t_{1}\right)\right)+W\right)\;+\varepsilon^{2}R_{0}\ln\left(E_{0}-\tanh^{2}\left(l_{1}\psi^{2b-1}+\Xi \frac{\partial L_{1}^{*}}{\partial \Gamma }\right)\right)=0$$

Furthermore, (17) can be written as

(18) $$\begin{array}{cc} V^{*} & =2\varepsilon^{2}\left(1-e^{-\chi t_{0}}\right)l_{1}\psi^{2b-1}\;\tanh\left(l_{1}\psi^{2b-1}+\Xi \frac{\partial L_{1}^{*}}{\partial \Gamma }\right)\\ & +\frac{\partial L_{1}^{*}}{\partial \Gamma }\left[F\left(\psi \right)+H\left(\psi \right)+W-\left(H\left(\psi \right)-H\left(\psi \left(t-t_{1}\right)\right)\right)\right] \end{array}\;-\chi L_{1}^{*}+\left(1-e^{-\chi t_{0}}\right)\Gamma^{T}Q\Gamma +\varepsilon^{2}R_{0}\ln\left(E_{0}-\tanh^{2}\left(l_{1}\psi^{2b-1}+\Xi \frac{\partial L_{1}^{*}}{\partial \Gamma }\right)\right)=0$$

To deal with the challenges brought by online tracking control, the optimal value $L_{1}^{*}$ should be solved using (17). Furthermore, the optimal control policy $u(L_{1}^{*})$ is shown in (14).

### 3.3. State Time Delay

Choosing the appropriate LKFs solves the problem caused by the state time delay, which laid the foundation for the application of the IRL algorithm.

According to Assumption 2 in [36] and Remark 5 in [15], the IRL method can be used to solve the $L_{1}^{*}$, only when the function $\Theta_{1}\left(t\right)=F\left(\psi \right)+H\left(\psi \right)$, $\Theta_{2}\left(t\right)=H\left(\psi \right)-H\left(\psi \left(t-t_{1}\right)\right)$ and G satisfy that

(19) $$\left\|F\left(\psi \right)+H\left(\psi \right)\right\|\leq b_{1}\left\|\psi \right\|$$

(20) $$\left\|H\left(\psi \right)-H\left(\psi \left(t-t_{1}\right)\right)\right\|\leq \sum_{\theta =1}^{n}b_{2,\theta }\left\|\psi \left(t-\theta \Delta t\right)\right\|$$

(21) $$\left\|G\right\|\leq b_{3}$$

where $b_{1}$, $b_{2,\theta }$, and $b_{3}$ are positive constants, with 0<θ≤n and $\Delta t=t_{1}/n$.

Considering the function $F\left(\psi \right)$ and the known function $H\left(\psi \right)$ satisfy the Lipchitz condition, and Assumption 2, (19) and (21) can be guaranteed. However, the state time delay $t_{1}$ is uncertain, and the boundedness of (20) cannot be obtained. In addition, because of the uncertain state time delay, the uncertain function $H\left(\psi \left(t-t_{1}\right)\right)$ cannot be approximated using NN.

In order to better complete the controller design, the problem caused by the state time delay will be handled first. Defining the new function $\Theta_{2}\left(t\right)$ as

(22) $$\Theta_{2}\left(t\right)=H\left(\psi \right)-H\left(\psi \left(t-t_{1}\right)\right)$$

and (22) can be written in the following form

(23) $$\begin{array}{cc} \Theta_{2}\left(t\right)= & H\left(\psi \right)-H\left(\psi \left(t-\Delta t\right)\right)+H\left(\psi \left(t-\Delta t\right)\right)\\ & -H\left(\psi \left(t-2\Delta t\right)\right)+H\left(\psi \left(t-2\Delta t\right)\right)-H\left(\psi \left(t-3\Delta t\right)\right)+\ldots \end{array}\;-H\left(\psi \left(t-\theta \Delta t\right)\right)+H\left(\psi \left(t-\left(\theta +1\right)\Delta t\right)\right)-\ldots \;+H\left(\psi \left(t-\left(n-1\right)\Delta t\right)\right)-H\left(\psi \left(t-t_{1}\right)\right)$$

where $\Delta t=t_{1}/n$, and both i and n are positive integers.

By Assumption 1, the mean-value theorem is introduced to $H\left(\psi \right)$. Therefore, one obtains

(24) $$\Delta \psi \left(t-\Delta t\right)=H\left(\psi \left(t\right)\right)-H\left(\psi \left(t-\Delta t\right)\right)={\left.\frac{\partial H\left(\psi \right)}{\partial \psi }\right|}_{\psi =\psi^{\eta }}\left(\psi \left(t\right)-\psi \left(t-\Delta t\right)\right)$$

where $\psi^{\eta }=\eta \psi \left(t\right)+\left(1-\eta \right)\psi \left(t-\Delta t\right)$, 0<η<1.

The error function caused by Δt can be obtained as

(25) $$\Delta \psi \left(t-\left(\theta +1\right)\Delta t\right)={\left.\frac{\partial H\left(\psi \right)}{\partial \psi }\right|}_{\psi =\psi^{\eta }}\left(\psi \left(t-\theta \Delta t\right)-\psi \left(t-\left(\theta +1\right)\Delta t\right)\right)$$

Defining the augmented system states as

(26) $$\Delta \bar{\psi }=\left[\Delta \psi \left(t\right),\ldots ,\Delta \psi \left(t-\theta \Delta t\right)\ldots ,\Delta \psi \left(t-t_{1}\right)\right]$$

then, we can write system (24) as follows

(27) $$\Delta \bar{\dot{\psi }}\left(t\right)=\Pi \left(\Delta \bar{\psi }\left(t\right)\right)$$

To guarantee that system (27) is uniformly ultimately bounded (UUB), the following lemma is proposed.

**Lemma** **3.** *If the dimension of the state vector matches that of the function* $\Pi \left(\Delta \psi \right)$*, where* $\Pi \left(\Delta \psi \left(0\right)\right)=0$.

(28) $$\Delta \dot{\psi }\left(t\right)=\Pi \left(\Delta \psi \left(t\right)\right)$$

*converges to a compact set exponentially, where the Lyapunov function satisfies*

(29) $$c_{1}{\left\|\Delta \psi \left(t\right)\right\|}^{2}\leq L_{0}\left(\Delta \psi \left(t\right)\right)\leq c_{2}{\left\|\Delta \psi \left(t\right)\right\|}^{2}+c_{3}$$

(30) $${\dot{L}}_{0}\left(\Delta \psi \left(t\right)\right)\leq -c_{4}{\left\|\Delta \psi \left(t\right)\right\|}^{2}+c_{5}$$

*where $c_{i}>0$, θ=1,2,…,5. In addition, the uniformly ultimately boundedness of system (28) can be guaranteed.*

**Proof.**  Inspired by the research in [26], the following proof process is given. Defining the initial state of (28) as $\Delta \dot{\psi }\left(0\right)=\Delta \psi \left(t\right)$, we obtain

(31) $$\left\{\begin{array}{l} \Delta \dot{\psi }\left(t\right)=\Pi \left(\Delta \dot{\psi }\left(t-\Delta t\right)\right)=\Pi \left(\Delta \psi \left(t\right)\right)\\ \Delta \dot{\psi }\left(t-\Delta t\right)=\Pi \left(\Delta \dot{\psi }\left(t-2\Delta t\right)\right)=\Pi^{2}\left(\Delta \psi \left(t\right)\right)\\ \;\;\;\vdots \\ \Delta \dot{\psi }\left(t-\theta \Delta t\right)=\Pi \left(\Delta \dot{\psi }\left(t-\left(n-\theta -1\right)\Delta t\right)\right)=\Pi^{\theta +1}\left(\Delta \psi \left(t\right)\right)\\ \;\;\;\vdots \\ \Delta \dot{\psi }\left(t-\left(n-1\right)\Delta t\right)=\Pi \left(\Delta \dot{\psi }\left(t-t_{1}\right)\right)=\Pi^{n}\left(\Delta \psi \left(t\right)\right) \end{array}\right.$$

If the system exponentially converges to a compact set, then

(32) $$\left\|\Pi^{\theta }\left(\Delta \psi \left(t\right)\right)\right\|\leq a_{1}a_{2}^{\theta }\left\|\Delta \psi \left(t\right)\right\|+a_{3}$$

where positive constants satisfy $a_{1}>0$, $0<a_{2}<1$, and $a_{3}\geq 0$.

Furthermore, the Lyapunov function is

(33) $$\Delta L_{0}=\sum_{i=0}^{n-1}{\left[\Pi^{\theta }\left(\Delta \psi \left(t\right)\right)\right]}^{T}\left[\Pi^{\theta }\left(\Delta \psi \left(t\right)\right)\right].$$

Submitting (32) to (33), we obtain

(34) $$\Delta L_{0}\leq 2a_{1}^{2}\sum_{i=0}^{n-1}a_{2}^{2\theta }{\left\|\Delta \psi \left(t\right)\right\|}^{2}+2na_{3}^{2}\leq 2a_{1}^{2}\sum_{i=0}^{n-1}{\left\|\Delta \psi \left(t\right)\right\|}^{2}+2na_{3}^{2}.$$

Considering the fact that $0<a_{2}<1$, we obtain

(35) $$c_{1}\sum_{i=0}^{n-1}{\left\|\Delta \psi \left(t\right)\right\|}^{2}\leq \Delta L_{0}\left(\Delta \psi \left(t\right)\right)\leq c_{2}\sum_{i=0}^{n-1}{\left\|\Delta \psi \left(t\right)\right\|}^{2}+c_{3}$$

where $c_{1}=1$, $c_{2}=2a_{1}^{2}\frac{1-a_{2}^{2n}}{1-a_{2}^{2}}$, $c_{3}=2na_{3}^{2}$.

Moreover, we can obtain

(36) $$\Delta {\dot{L}}_{0}\left(\Delta \psi \left(t\right)\right)=\frac{1}{\Delta t}\left[\Delta L_{0}\left(\Delta \psi \left(t+\Delta t\right)\right)-\Delta L_{0}\left(\Delta \psi \left(t\right)\right)\right]\;=\frac{1}{\Delta t}\left[\sum_{i=1}^{n}{\left[\Pi^{i}\left(\Delta \psi \left(t\right)\right)\right]}^{2}-\sum_{i=0}^{n-1}{\left[\Pi^{i}\left(\Delta \psi \left(t\right)\right)\right]}^{2}\right]\;\leq \frac{1}{\Delta t}\left[{\left[a_{1}a_{2}^{n}\left\|\Delta \psi \left(t\right)\right\|+a_{3}\right]}^{2}-{\left\|\Delta \psi \left(t\right)\right\|}^{2}\right]\;\leq -\left(1-2a_{1}^{2}a_{2}^{2n}\right)\frac{1}{\Delta t}{\left\|\Delta \psi \left(t\right)\right\|}^{2}+2a_{3}^{2}\frac{1}{\Delta t}\;\leq -c_{4}{\left\|\Delta \psi \left(t\right)\right\|}^{2}+c_{5}$$

where $c_{4}=\left(1-2a_{1}^{2}a_{2}^{2n}\right)/\Delta t$ and $c_{5}=2a_{3}^{2}/\Delta t$. If a sufficiently small-time interval Δt is chosen, n will be sufficiently large to ensure that $c_{4}$ is a positive constant.

Based on, (35) and (36), one has

(37) $$\begin{array}{cc} \Delta {\dot{L}}_{0}\left(\Delta \psi \left(t\right)\right) & \leq -\left(1-2a_{1}^{2}a_{2}^{2n}\right){\left\|\Delta \psi \left(t\right)\right\|}^{2}+2a_{3}^{2}\\ & \leq -\frac{\left(1-2a_{1}^{2}a_{2}^{2n}\right)\left(1-a_{2}^{2}\right)}{2a_{1}^{2}\left(1-a_{2}^{2n}\right)}L_{0}\left(\Delta \psi \left(t\right)\right)\\ & \;+\frac{2\left(1-a_{2}^{2}\right)\left(1-2a_{1}^{2}a_{2}^{2n}\right)na_{3}^{2}}{2a_{1}^{2}\left(1-a_{2}^{2n}\right)}+2a_{3}^{2}\\ & =-b_{1}\Delta L_{0}\left(\Delta \psi \left(t\right)\right)+b_{2} \end{array}$$

where

(38) $$b_{1}=\frac{\left(1-2a_{1}^{2}a_{2}^{2n}\right)\left(1-a_{2}^{2}\right)}{2a_{1}^{2}\left(1-a_{2}^{2n}\right)}$$

(39) $$b_{2}=\frac{2\left(1-a_{2}^{2}\right)\left(1-2a_{1}^{2}a_{2}^{2n}\right)na_{3}^{2}}{2a_{1}^{2}\left(1-a_{2}^{2n}\right)}+2a_{3}^{2}$$

Furthermore, we can obtain the Lyapunov function (23), and guarantee the UUB of system (23), which is composed by *n* subsystems similar to (28)

(40) $${\dot{L}}_{0}\left(\Delta \psi \left(t\right)\right)=\sum_{i=1}^{n}\Delta {\dot{L}}_{0}\left(\Delta \psi \left(t-\theta \Delta t\right)\right)$$

Substituting (36) for (40), one has

(41) $${\dot{L}}_{0}\left(\Delta \psi \left(t\right)\right)\leq -c_{4}\sum_{i=1}^{n}{\left\|\Delta \psi \left(t-\theta \Delta t\right)\right\|}^{2}+c_{5}$$

Similarly, one has

(42) $${\dot{L}}_{0}\left(\Delta \psi \left(t\right)\right)\leq -b_{1}L_{0}\left(\Delta \psi \left(t\right)\right)+b_{2}$$

when n and $a_{1}$ are selected large enough, the ultimate boundedness of $L_{0}\left(\Delta \psi \left(t\right)\right)$ can be assured for any initial condition $L_{0}\left(\Delta \psi \left(t-t_{1}\right)\right)$ within a bounded set, guaranteeing the UUB of system states are guaranteed.

The proof is competed. □

### 3.4. Critic NN and Value Function Approximation

In summary, the boundedness of (19)–(21) can be obtained. The IRL method will be extended to the solution of $L_{1}^{*}$ in the following section.

When the IRL interval is choose as T>0, (8) can be written as the following form

(43) $$L_{1}\left(t-T\right)=\left(1-e^{-\chi t_{0}}\right)\int_{t-T}^{t}e^{-\chi \left(\tau -\left(t-T\right)\right)}\left(\Gamma^{T}Q\Gamma +U\left(u\right)\right)d\tau +e^{-\chi T}L_{1}\left(t\right)$$

Assuming that (8) is a continuous smooth function, $L_{1}$ and its gradient $\partial L_{1}/\partial \psi$ are approximated as

(44) $$L_{1}=\omega_{c}^{T}\varphi_{c}\left(\Gamma \right)+\varepsilon_{c}$$

(45) $$\frac{\partial L_{1}}{\partial \Gamma }=\omega_{c}\frac{\partial \varphi_{c}^{T}}{\partial \Gamma }+\frac{\partial \varepsilon_{c}}{\partial \Gamma }$$

where $\omega_{c}\in {\mathbb{R}}^{l_{c}}$ is the constant-target online estimate parameter vector, in which $l_{c}$ is the quantity of neurons within the neural network, and $\varphi_{c}$ and $\varepsilon_{c}$ are the activation function of the critic NN and approximate error, respectively.

**Assumption** **4.** *The boundedness of the activation function and assessment of the error of the critic NN and their gradient can be obtained as* $\left\|\varphi_{c}\right\|\leq {\bar{\varphi }}_{c}$*, and* $\left\|\varepsilon_{c}\right\|\leq {\bar{\varepsilon }}_{c}$*,* $\left\|\partial \varphi_{c}/\partial \Psi \right\|\leq {\bar{\varepsilon }}_{c,0}$ *and* $\left\|\partial \varepsilon_{c}/\partial \Psi \right\|\leq {\bar{\varepsilon }}_{c,0}$*, respectively.*

When the IRL interval is T>0, the Bellman equation induced in the critic NN estimated value, can be expressed as

(46) $$z_{B}=\int_{t-T}^{t}e^{-\chi \left(\tau -t+T\right)}\left(1-e^{-\chi t_{0}}\right)\left[\Gamma^{T}Q\Gamma +U\left(u\right)\right]d\tau +\omega_{c}^{T}\Delta \varphi_{c}$$

where

(47) $$\Delta \varphi_{c}=e^{-\chi T}\varphi_{c}\left(\Gamma \left(t\right)\right)-\varphi_{c}\left(\Gamma \left(t-T\right)\right)$$

The constraint on (46) can be derived based on Assumption 4, i.e., $\left\|z_{B}\right\|\leq {\bar{z}}_{B}$.

To derive the approximate tracking Bellman function, the approximation of the neural network is evaluated to obtain

(48) $${\hat{L}}_{1}={\hat{\omega }}_{c}^{T}\varphi_{c}\left(\Gamma \right)$$

where ${\hat{\omega }}_{c}$ is the estimation of the critic law $\omega_{c}$.

Therefore, the estimation of (46) is

(49) $$z_{B}=R\left(t\right)+{\hat{\omega }}_{c}^{T}\Delta \varphi_{c}$$

where the reinforcement learning reward is denoted by

(50) $$r\left(t\right)=\int_{t-T}^{t}e^{-\chi \left(\tau -t+T\right)}\left(1-e^{-\chi t_{0}}\right)\left[\Gamma^{T}Q\Gamma +\hat{U}\left(u\right)\right]d\tau .$$

To reduce the approximation error, we give a function in (51) from

(51) $$Z_{B}\left(t\right)=1/2z_{B}^{2}\left(t\right).$$

We can obtain the following expression using the Gradient descent method.

(52) $${\dot{\hat{\omega }}}_{c}=\alpha_{c}\frac{\Delta \varphi_{c}\Delta \varphi_{c}^{T}}{{\left\|1+\Delta \varphi_{c}^{T}\Delta \varphi_{c}\right\|}^{2}}z_{B}$$

where $\alpha_{c}$ represents the learning rate of the critic neural network.

Considering ${\tilde{\omega }}_{c}=\omega_{c}-{\hat{\omega }}_{c}$, (46) and (49), we have

(53) $$z_{B}={\tilde{\omega }}_{c}^{T}\Delta \varphi_{c}+\varepsilon_{B}$$

(54) $${\dot{\tilde{\omega }}}_{c}=-\alpha_{c}\frac{\Delta \varphi_{c}\Delta \varphi_{c}^{T}}{{\left\|1+\Delta \varphi_{c}^{T}\Delta \varphi_{c}\right\|}^{2}}{\tilde{\omega }}_{c}+\alpha_{c}\frac{\Delta \varphi_{c}\Delta \varphi_{c}^{T}}{{\left\|1+\Delta \varphi_{c}^{T}\Delta \varphi_{c}\right\|}^{2}}\varepsilon_{B}$$

### 3.5. Action NN and Controller Design

According to (45), the optimal control input, i.e., (14) is as follows

(55) $$u=-\varepsilon \;\tanh\left[l_{1}\psi^{2b-1}+\Xi \left(\omega_{c}\frac{\partial \varphi_{c}^{T}}{\partial \Gamma }+\frac{\partial \varepsilon_{c}}{\partial \Gamma }\right)\right]$$

To solve the issue in the tracking HJB induced $\frac{\partial \varepsilon_{c}}{\partial \Psi }$, we obtain

(56) $$\int_{t-T}^{t}e^{-\chi \left(v-t+T\right)}{\dot{\varphi }}_{c}dv=\int_{t-T}^{t}e^{-\chi \left(v-t+T\right)}\frac{\partial \varphi_{c}}{\partial \Gamma }\left(\Theta_{1}+\Theta_{2}+Gu+W\right)dv\;=\Delta \varphi_{c}+\chi \int_{t-T}^{t}e^{-\chi \left(v-t-T\right)}\varphi_{c}dv$$

In addition, we obtain

(57) $$\Delta \varphi_{c}=\int_{t-T}^{t}e^{-\chi \left(v-t+T\right)}\left(\frac{\partial \varphi_{c}}{\partial \Gamma }\left(\Theta_{1}+\Theta_{2}+Gu+W\right)-\chi \varphi_{c}\right)dv$$

Equation (16) becomes

(58) $$U\left(u\right)=\varepsilon^{2}R_{0}\ln\left\{E_{0}-\tanh^{2}\left[l_{1}\psi^{2b-1}+\Xi \left(\omega_{c}^{T}\frac{\partial \varphi_{c}}{\partial \Gamma }+\frac{\partial \varepsilon_{c}}{\partial \Gamma }\right)\right]\right\}\;-2\varepsilon \left(l_{1}\psi^{2b-1}+\Xi \left(\omega_{c}^{T}\frac{\partial \varphi_{c}}{\partial \Gamma }+\frac{\partial \varepsilon_{c}}{\partial \Gamma }\right)\right)u$$

Then, (46) can be rewritten as

(59) $$\begin{array}{l} \int_{t-T}^{t}e^{-\chi \left(\tau -t+T\right)}\left\{\left(1-e^{-\chi t_{1}}\right)\Gamma^{T}Q\Gamma -\chi \omega_{c}^{T}\varphi_{c}\right.\\ +2\varepsilon^{2}\left(1-e^{-\chi t_{1}}\right)l_{1}\psi^{2b-1}\;\tanh\left[l_{1}\psi^{2b-1}+\Xi \omega_{c}\frac{\partial \varphi_{c}^{T}}{\partial \Gamma }\right]+\varepsilon_{HJB}\\ +\omega_{c}\frac{\partial \varphi_{c}^{T}}{\partial \Gamma }\left(\Theta_{1}+\Theta_{2}+W\right)\left.+\varepsilon^{2}R_{0}\ln\left\{E_{0}-\tanh^{2}\left[l_{1}\psi^{2b-1}+\Xi \left(\omega_{c}\frac{\partial \varphi_{c}^{T}}{\partial \Gamma }\right)\right]\right\}\right\}dv=0 \end{array}$$

where

(60) $$\varepsilon_{HJB}=e^{-\chi \left(\tau -t+T\right)}\left\{-\varepsilon^{2}R_{0}\ln\left[E_{0}-\tanh^{2}\left(l_{1}\psi^{2b-1}+\Xi \omega_{c}\frac{\partial \varphi_{c}^{T}}{\partial \Gamma }\right)\right]\right.-2\varepsilon^{2}\left(1-e^{-\chi t_{0}}\right)l_{1}\psi^{2b-1}\;\;\;\times \tanh\left[l_{1}\psi^{2b-1}+\Xi \omega_{c}\frac{\partial \varphi_{c}^{T}}{\partial \Gamma }\right]+2\varepsilon^{2}\left(1-e^{-\chi t_{0}}\right)l_{1}\psi^{2b-1}\;\tanh\left[l_{1}\psi^{2b-1}+\Xi \left(\omega_{c}\frac{\partial \varphi_{c}^{T}}{\partial \Gamma }+\frac{\partial \varepsilon_{c}}{\partial \Gamma }\right)\right]\;\left.-\omega_{c}^{T}\frac{\partial \varepsilon_{c}}{\partial \Gamma }\left(\Theta_{1}+\Theta_{2}+W\right)+\varepsilon^{2}R_{0}\ln\left[E_{0}-\tanh^{2}\left(l_{1}\psi^{2b-1}+\Xi \left(\omega_{c}\frac{\partial \varphi_{c}^{T}}{\partial \Gamma }+\frac{\partial \varepsilon_{c}}{\partial \Gamma }\right)\right)\right]\right\}$$

To obtain the limitation of the HJB proximity error, we can use the boundary proximity error. In addition, when NN is selected, the construction cannot be changed. We can only solve this problem by uncertain weights of NN.

Approximating the control input (55) by critic NN, we have

(61) $$u_{1}=-\varepsilon \;\tanh\left(l_{1}\psi^{2b-1}+\Xi {\hat{\omega }}_{c}\frac{\partial \varphi_{c}^{T}}{\partial \Gamma }\right)$$

where ${\hat{\omega }}_{c}$ is the estimated value of $\omega_{c}$.

However, the function of (61) is only to estimate the current critical NN weight, which fails to keep the system (1) stable. Hence, to guarantee the stability of the system and solve the optimal control strategy, we introduce another NN as the action NN.

(62) $${\hat{u}}_{1}=-\varepsilon \;\tanh\left(l_{1}\psi^{2b-1}+\Xi {\hat{\omega }}_{a}\frac{\partial \varphi_{c}^{T}}{\partial \Gamma }\right)$$

where ${\hat{\omega }}_{a}$ represents the weight vector of the action neural network, denoting the present evaluation value of $\omega_{c}$.

Then, the interval IRL Bellman equation error is estimated as

(63) $${\hat{z}}_{B}=\int_{t-T}^{t}e^{-\chi \left(\tau -t+T\right)}\left[\Gamma^{T}Q\Gamma +\hat{U}\left(u\right)\right]d\tau +{\hat{\omega }}_{c}^{T}\Delta \varphi_{c}$$

where

(64) $$\hat{U}\left(u\right)=2\int_{0}^{\hat{u}}{\left(\varepsilon {\;\tanh}^{-1}\left(v/\varepsilon \right)\right)}^{T}Rd_{v}$$

Therefore, (52) can be rewritten as

(65) $${\dot{\hat{\omega }}}_{c}=\alpha_{c}\frac{\Delta \varphi_{c}\Delta \varphi_{c}^{T}}{{\left\|1+\Delta \varphi_{c}^{T}\Delta \varphi \right\|}^{2}}{\hat{z}}_{B}$$

defining the input assessment error as

(66) $$z_{u}={\hat{u}}_{1}-u_{1}=\varepsilon \left(\tanh\left(l_{1}\psi^{2b-1}+\Xi {\hat{\omega }}_{a}\frac{\partial \varphi_{a}^{T}}{\partial \Gamma }\right)-\tanh\left(l_{1}\psi^{2b-1}+\Xi {\hat{\omega }}_{c}\frac{\partial \varphi_{c}^{T}}{\partial \Gamma }\right)\right)$$

To minimize (66), we use the following formula

(67) $$Z_{u}\left(t\right)=z_{u}^{T}\left(t\right)Rz_{u}\left(t\right).$$

We can utilize the gradient descent method to derive the following equation

(68) $${\dot{\hat{\omega }}}_{a}=-\alpha_{a}\left[\Xi^{\prime }\frac{\partial \varphi_{a}^{T}}{\partial \Gamma }{\mathrm{sech}}^{2}\left(l_{1}\psi^{2b-1}+\Xi \frac{\partial \varphi_{a}^{T}}{\partial \Gamma }{\hat{\omega }}_{a}\right)z_{u}+\eta {\hat{\omega }}_{a}\right]$$

where $\Xi^{\prime }=R\Xi$, and η is a positive design variable.

### 3.6. Stability Analysis

According to the proposed lemmas and assumptions, the following theorem is given to analyze the effectiveness of the proposed algorithm.

**Theorem** **1.** *Based on the definition in [43], Lemma 1–4, Assumptions 1–4, and the design of the proposed control policy (62) and the control laws, (65) and (68), the proposed optimal tracking control algorithm ensures that the partially uncertain nonlinear system (1) is SGPFS.*

**Proof** **of** **Theorem** **1.** The candidate function of the Lyapunov function is designed as

(69) $$L\left(k\right)=L_{0}\left(k\right)+L_{1}\left(k\right)+L_{2}\left(k\right)+L_{3}\left(k\right)+L_{4}\left(k\right)$$

where ${\dot{L}}_{0}\left(\Delta \psi \left(t\right)\right)\leq -c_{4}\sum_{i=1}^{n}{\left\|\Delta \psi \left(t-\theta \Delta t\right)\right\|}^{2}+c_{5}$ and $L_{1}\left(k\right)$ is given in (8) as the optimal value function. Then, the provided expressions are applicable:

(70) $$L_{2}=\sum_{i=1}^{n}{\left(\Delta \psi \left(t-\theta \Delta t\right)\right)}^{T}Q_{3}\left(\Delta \psi \left(t-\theta \Delta t\right)\right)$$

(71) $$L_{3}=1/\alpha_{c}{\tilde{\omega }}_{c}^{T}{\tilde{\omega }}_{c}$$

(72) $$L_{4}=1/\alpha_{a}{\tilde{\omega }}_{a}^{T}{\tilde{\omega }}_{a}$$

Based on (24) and (31), the first derivative of $L_{2}$ can be given as

(73) $${\dot{L}}_{2}=2\sum_{j=1}^{n}\Delta \psi \left(t-i\Delta t\right)Q_{3}\Delta \dot{\psi }\left(t-i\Delta t\right)\;=2{\left(\Delta \psi \left(t-n\Delta t\right)\right)}^{T}Q_{3}\left(\Delta \psi \left(t\right)\right)+\ldots +{\left(\Delta \psi \left(t-\Delta t\right)\right)}^{T}Q_{3}\left(\Delta \psi \left(t-\left(n-1\right)\Delta t\right)\right)\;+{\left(\Delta \psi \left(t\right)\right)}^{T}Q_{3}\left(\Delta \psi \left(t-n\Delta t\right)\right)$$

Using Young’s inequality $\pm a^{T}b_{0}\leq \mu a^{T}a/2+{b_{0}}^{T}b_{0}/\left(2\mu \right)$, one has

(74) $$\begin{array}{cc} {\dot{L}}_{2} & \leq \mu {\left(\Delta \psi \left(t-n\Delta t\right)\right)}^{T}Q_{3}\left(\Delta \psi \left(t-n\Delta t\right)\right)+\frac{1}{\mu }{\left(\Delta \psi \left(t\right)\right)}^{T}Q_{3}\left(\Delta \psi \left(t\right)\right)+\ldots \\ & +\mu {\left(\Delta \psi \left(t-\Delta t\right)\right)}^{T} \end{array}\;\begin{array}{l} \;\times Q_{3}\left(\Delta \psi \left(t-\Delta t\right)\right)+\frac{1}{\mu }{\left(\Delta \psi \left(t-\left(n-1\right)\Delta t\right)\right)}^{T}Q_{3}\left(\Delta \psi \left(t-\left(n-1\right)\Delta t\right)\right)\\ \;+\mu {\left(\Delta \psi \left(t\right)\right)}^{T}Q_{3}\left(\Delta \psi \left(t\right)\right) \end{array}\;+\frac{1}{\mu }{\left(\Delta \psi \left(t-n\Delta t\right)\right)}^{T}Q_{3}\left(\Delta \psi \left(t-n\Delta t\right)\right)$$

(75) $${\dot{L}}_{2}\leq c_{0}\sum_{i=1}^{n}{\left\|\Delta \psi \left(t-\theta \Delta t\right)\right\|}^{2}$$

where $c_{0}=\left(\mu +1/\mu \right)Q_{3}$.

Based on (44), the first derivative of $L_{3}$ is

(76) $${\dot{L}}_{3}={\hat{\omega }}_{c}^{T}\frac{\partial \varphi }{\partial \Gamma }\left(\Theta_{1}+\Theta_{2}+Gu+W\right)$$

Considering (57), with the IRL interval chosen small enough, we have $\rho_{1}\varphi_{c}=\varphi_{c}\left(t-T\right)$, $\rho_{1}=1\pm \rho_{0}$, $\rho_{1}\in U\left(1,\rho_{0}\right)$, and $\rho_{0}$ is a sufficiently small positive constant.

(77) $$\left(\chi +\frac{1}{T}\right)\varphi -\frac{\varphi_{c}\left(t-T\right)}{Te^{-\chi T}}\approx \frac{\partial \varphi }{\partial \Gamma }\left(\Theta_{1}+\Theta_{2}+Gu+W\right)$$

Then, (76) can be written as

(78) $${\dot{L}}_{3}=\left(\chi +\frac{1}{T}\right){\hat{\omega }}_{c}^{T}\varphi -\frac{1}{Te^{-\chi T}}{\hat{\omega }}_{c}^{T}\varphi_{c}\left(t-T\right)\;\leq \left({\left(\chi +\frac{1}{T}\right)}^{2}+\frac{1}{T^{2}e^{-2\chi T}}\right){\left({\hat{\omega }}_{c}^{T}\varphi \right)}^{T}{\hat{\omega }}_{c}^{T}\varphi_{c}$$

Based on (54) we have

(79) $$\varepsilon_{B}=\frac{T}{t_{1}}{\hat{\omega }}_{c}^{T}\varphi_{c}+{\hat{\omega }}_{c}^{T}\left(e^{-\chi T}\varphi_{c}-\varphi_{c}\left(t-T\right)\right)$$

Then, the approximate of (54) is

(80) $${\dot{\tilde{\omega }}}_{c}=-\alpha_{c}\frac{\Delta \varphi \Delta \varphi^{T}}{{\left\|1+\Delta \varphi^{T}\Delta \varphi \right\|}^{2}}{\tilde{\omega }}_{c}\;+\alpha_{c}\frac{\Delta \varphi }{{\left\|1+\Delta \varphi^{T}\Delta \varphi \right\|}^{2}}\left(\frac{T}{t_{1}}{\hat{\omega }}_{c}^{T}\varphi_{c}+{\hat{\omega }}_{c}^{T}\left(e^{-\chi T}\varphi_{c}-\varphi_{c}\left(t-T\right)\right)\right)$$

And, for the first difference of (71)

(81) $${\dot{L}}_{3}=-\frac{\Delta \varphi_{c}\Delta \varphi_{c}^{T}}{{\left\|1+\Delta \varphi_{c}^{T}\Delta \varphi \right\|}^{2}}{\tilde{\omega }}_{c}^{T}{\tilde{\omega }}_{c}\;+\left(\omega_{c}^{T}-{\hat{\omega }}_{c}^{T}\right)\frac{\Delta \varphi_{c}}{{\left\|1+\Delta \varphi_{c}^{T}\Delta \varphi \right\|}^{2}}\left(\frac{T}{t_{1}}{\hat{\omega }}_{c}^{T}\varphi_{c}+{\hat{\omega }}_{c}^{T}\left(e^{-\chi T}\varphi_{c}-\varphi_{c}\left(t-T\right)\right)\right)$$

Using Cauchy’s mean value theorem, (81) changed into

(82) $${\dot{L}}_{3}=\frac{1}{{\left\|1+\Delta \varphi_{c}^{T}\Delta \varphi_{c}\right\|}^{2}}-\left[{\left\|\Delta \varphi \right\|}^{2}{\tilde{\omega }}_{c}^{T}{\tilde{\omega }}_{c}^{T}+{\left(\omega_{c}^{T}\Delta \varphi_{c}\right)}^{T}\omega_{c}^{T}\Delta \varphi_{c}\right.\;\left.-\frac{1}{2}\left(\frac{T}{t_{1}}\left(e^{-\chi T}-\rho_{1}\right)-\frac{T^{2}}{t_{1}^{2}}\right){\left({\hat{\omega }}_{c}^{T}\varphi_{c}\right)}^{T}{\hat{\omega }}_{c}^{T}\varphi_{c}\right]$$

Based on (68) and (66), we have

(83) $${\dot{\tilde{\omega }}}_{a}=\alpha_{a}\left[\Xi^{\prime }\frac{\partial \varphi_{a}}{\partial \Gamma }{\mathrm{sech}}^{2}\left(l_{1}\psi^{2b-1}+\Xi^{T}\frac{\partial \varphi_{c}^{T}}{\partial \Gamma }{\hat{\omega }}_{a}\right)\times \right.\;\left(\tanh\left(\Xi {\hat{\omega }}_{a}\frac{\partial \varphi_{a}^{T}}{\partial \Gamma }+l_{1}\psi^{2b-1}\right)-\tanh\left(l_{1}\psi^{2b-1}+\Xi {\hat{\omega }}_{c}\frac{\partial \varphi_{c}^{T}}{\partial \Gamma }\right)\right)\left.-\eta {\tilde{\omega }}_{a}+\eta \omega_{c}\right]$$

Then, the first derivative of $L_{3}$ is

(84) $${\dot{L}}_{4}=-\eta {\tilde{\omega }}_{a}^{T}{\tilde{\omega }}_{a}+{\tilde{\omega }}_{a}^{T}J_{0}$$

where

(85) $$J_{0}=\Xi^{\prime }\frac{\partial \varphi_{a}}{\partial \Gamma }{\mathrm{sech}}^{2}\left(l_{1}\psi^{2b-1}+\Xi^{T}\frac{\partial \varphi_{a}^{T}}{\partial \Gamma }{\hat{\omega }}_{a}\right)\left[\left(\tanh\left(\Xi {\hat{\omega }}_{a}\frac{\partial \varphi_{a}^{T}}{\partial \Gamma }+l_{1}\psi^{2b-1}\right)\right.\right.\;\left.\left.-\tanh\left(l_{1}\psi^{2b-1}+\Xi {\hat{\omega }}_{c}\frac{\partial \varphi_{c}^{T}}{\partial \Gamma }\right)\right)+\eta \omega_{c}\right]$$

By using Cauchy’s mean value theorem, we have

(86) $${\dot{L}}_{4}\leq -\left(\eta -1/\left(2\alpha_{a}\right)\right){\tilde{\omega }}_{a}^{T}{\tilde{\omega }}_{a}+\frac{\alpha_{a}}{2}J_{0}^{2}$$

Above all, the first difference of (69)

(87) $$\dot{L}\leq -c_{1}{\left({\hat{\omega }}_{c}^{T}\varphi \right)}^{T}{\hat{\omega }}_{c}^{T}\varphi -c_{2}{\tilde{\omega }}_{c}^{T}{\tilde{\omega }}_{c}-c_{3}{\tilde{\omega }}_{a}^{T}{\tilde{\omega }}_{a}-c_{6}\sum_{i=1}^{n}{\left\|\Delta \psi \left(t-\theta \Delta t\right)\right\|}^{2}+J^{2}$$

where $c_{\theta }>0$, θ=1,2,3, $c_{1}=\left(\frac{\frac{T}{t_{1}}\left(e^{-\chi T}-\rho_{1}\right)-\frac{T^{2}}{t_{1}^{2}}}{2{\left\|1+\Delta \varphi_{c}^{T}\Delta \varphi_{c}\right\|}^{2}}-{\left(\chi +\frac{1}{T}\right)}^{2}+\frac{1}{T^{2}e^{-2\chi T}}\right)$, $c_{2}=\frac{{\left\|\Delta \varphi_{c}\right\|}^{2}}{{\left\|1+\Delta \varphi_{c}^{T}\Delta \varphi_{c}\right\|}^{2}}$, $c_{3}=\left(\eta -\frac{1}{2\alpha_{a}}\right)$, $c_{6}=c_{4}-c_{0}$ and $J^{2}=\frac{{\left(\omega_{c}^{T}\Delta \varphi_{c}\right)}^{T}\omega_{c}^{T}\Delta \varphi_{c}}{{\left\|1+\Delta \varphi_{c}^{T}\Delta \varphi_{c}\right\|}^{2}}+c_{5}+\frac{\alpha_{a}}{2}J_{0}^{2}$.

To make the finite-time convergence, we deal with the equation and add and subtract several terms on the right side

(88) $$\begin{array}{cc} \dot{L} & \leq -c_{1}{\left({\left({\hat{\omega }}_{c}^{T}\varphi_{c}\right)}^{T}{\hat{\omega }}_{c}^{T}\varphi_{c}\right)}^{\beta }+c_{1}{\left({\left({\hat{\omega }}_{c}^{T}\varphi_{c}\right)}^{T}{\hat{\omega }}_{c}^{T}\varphi_{c}\right)}^{\beta }\\ & -c_{1}{\left({\hat{\omega }}_{c}^{T}\varphi_{c}\right)}^{T}{\hat{\omega }}_{c}^{T}\varphi_{c}-c_{2}{\left({\tilde{\omega }}_{c}^{T}{\tilde{\omega }}_{c}\right)}^{\beta }+c_{2}{\left({\tilde{\omega }}_{c}^{T}{\tilde{\omega }}_{c}\right)}^{\beta } \end{array}\;-c_{2}{\tilde{\omega }}_{c}^{T}{\tilde{\omega }}_{c}-c_{3}{\left({\tilde{\omega }}_{a}^{T}{\tilde{\omega }}_{a}\right)}^{\beta }+c_{3}{\left({\tilde{\omega }}_{a}^{T}{\tilde{\omega }}_{a}\right)}^{\beta }-c_{3}{\tilde{\omega }}_{a}^{T}{\tilde{\omega }}_{a}-c_{6}{\left(\sum_{i=1}^{n}{\left\|\Delta \psi \left(t-\theta \Delta t\right)\right\|}^{2}\right)}^{\beta }\;+c_{6}{\left(\sum_{i=1}^{n}{\left\|\Delta \psi \left(t-\theta \Delta t\right)\right\|}^{2}\right)}^{\beta }-c_{6}\sum_{i=1}^{n}{\left\|\Delta \psi \left(t-\theta \Delta t\right)\right\|}^{2}+J^{2}$$

To make the system (1) stable, and the finite time, we consider Lemma 1. Therefore, the constant must be greater than zero.

(89) $$\alpha_{a}>\frac{1}{2\eta }$$

and there are the following formulas:

(90) $$2{\left\|1+\Delta \varphi^{T}\Delta \varphi \right\|}^{2}\left({\left(\chi +\frac{1}{T}\right)}^{2}-\frac{1}{T^{2}e^{-2\gamma T}}\right)t_{1}^{2}-T\left(e^{-\chi T}-\rho_{1}\right)t_{1}+T^{2}<0$$

with

(91) $$e^{-\chi T}>\rho_{1}$$

(92) $$T\chi +1>\frac{1}{e^{-\chi T}}$$

then

(93) $$t_{1}>\frac{e^{-\chi T}-\rho_{1}+\sqrt{{\left(e^{-\chi T}-\rho_{1}\right)}^{2}-8{\left\|1+\Delta \varphi_{c}^{T}\Delta \varphi_{c}\right\|}^{2}\left({\left(\chi +\frac{1}{T}\right)}^{2}-\frac{1}{T^{2}e^{-2\chi T}}\right)}}{2{\left\|+1\Delta \varphi_{c}^{T}\Delta \varphi_{c}\right\|}^{2}\left({\left(\chi +\frac{1}{T}\right)}^{2}-\frac{1}{T^{2}e^{-2\chi T}}\right)}T.$$

Taking Lemma 1 to $c_{1}{\left({\hat{\omega }}_{c}^{T}\varphi_{c}\right)}^{T}{\hat{\omega }}_{c}^{T}\varphi_{c}$, $c_{2}{\tilde{\omega }}_{c}^{T}{\tilde{\omega }}_{c}$ and $c_{3}{\tilde{\omega }}_{a}^{T}{\tilde{\omega }}_{a}$, as x=1, and $y=c_{1}{\left({\hat{\omega }}_{c}^{T}\varphi_{c}\right)}^{T}{\hat{\omega }}_{c}^{T}\varphi_{c}$, or $y=c_{2}{\tilde{\omega }}_{c}^{T}{\tilde{\omega }}_{c}$, or $y=c_{3}{\tilde{\omega }}_{a}^{T}{\tilde{\omega }}_{a}$, or $y=c_{6}\sum_{i=1}^{n}{\left\|\Delta \psi \left(t-\theta \Delta t\right)\right\|}^{2}$ with $\mu_{1}=b$, $\mu_{2}=1-b$ and $l={\left(1-b\right)}^{\frac{1-b}{b}}$. Then, we have

(94) $$c_{1}{\left({\left({\hat{\omega }}_{c}^{T}\varphi_{c}\right)}^{T}{\hat{\omega }}_{c}^{T}\varphi_{c}\right)}^{b}\leq bl+c_{1}{\left({\hat{\omega }}_{c}^{T}\varphi_{c}\right)}^{T}{\hat{\omega }}_{c}^{T}\varphi_{c}$$

(95) $$c_{2}{\left({\tilde{\omega }}_{c}^{T}{\tilde{\omega }}_{c}\right)}^{b}\leq bl+c_{2}{\tilde{\omega }}_{c}^{T}{\tilde{\omega }}_{c}$$

(96) $$c_{3}{\left({\tilde{\omega }}_{a}^{T}{\tilde{\omega }}_{a}\right)}^{b}\leq bl+c_{3}{\tilde{\omega }}_{a}^{T}{\tilde{\omega }}_{a}$$

(97) $$c_{6}{\left(\sum_{i=1}^{n}{\left\|\Delta \psi \left(t-\theta \Delta t\right)\right\|}^{2}\right)}^{b}\leq bl+c_{6}\sum_{i=1}^{n}{\left\|\Delta \psi \left(t-\theta \Delta t\right)\right\|}^{2}.$$

Considering (94)–(97), (88) can be rewritten as

(98) $$\begin{array}{cc} \dot{L} & \leq -c_{1}{\left({\left({\hat{\omega }}_{c}^{T}\varphi_{c}\right)}^{T}{\hat{\omega }}_{c}^{T}\varphi_{c}\right)}^{b}-c_{2}\alpha_{c}^{-b}{\left({\tilde{\omega }}_{c}^{T}\alpha_{c}^{-1}{\tilde{\omega }}_{c}\right)}^{b}\\ & -c_{6}Q_{3}^{-1}{\left(\sum_{i=1}^{n}{\left(\Delta \psi \left(t-\theta \Delta t\right)\right)}^{T}Q_{3}\left(\Delta \psi \left(t-\theta \Delta t\right)\right)\right)}^{b} \end{array}\;-c_{3}\alpha_{a}^{-b}{\left({\tilde{\omega }}_{a}^{T}\alpha_{a}^{-1}{\tilde{\omega }}_{a}\right)}^{b}+4bl+J^{2}.$$

Inspired by reference [45], we have $\dot{\hat{x}}\left(t\right)=-\chi \hat{x}\left(t\right)+\kappa \upsilon \left(t\right)$, if $\upsilon \left(t\right)>0$, $\forall t>t_{0}$, $\dot{\hat{x}}>0$, $x\left(t\right)>0$, $\forall t>t_{0}$.

(99) $$\dot{L}\leq -cL^{b}+\pi$$

where

(100) $$c=\min\left(c_{1},c_{2}\alpha_{c}^{-b},-c_{3}\alpha_{a}^{-b},c_{6}Q_{3}^{-1}\right)$$

(101) $$\pi =4bl+J^{2}.$$

Based on (89)–(93), (99)–(101), and the lemma in [39], the boundedness of all singles in the closed loop nonstrict system is SGPFS for $\forall t>t_{1}$.

Furthermore, by using (62), we obtain the optimal control strategy, which guarantees that the target nonlinear system system’s state and the stability of input delays are maintained, and that the tracking error converges to a sufficiently small neighborhood around zero.

The proof is completed. □

## 4. Results of Simulation Example

**Figure 2:**
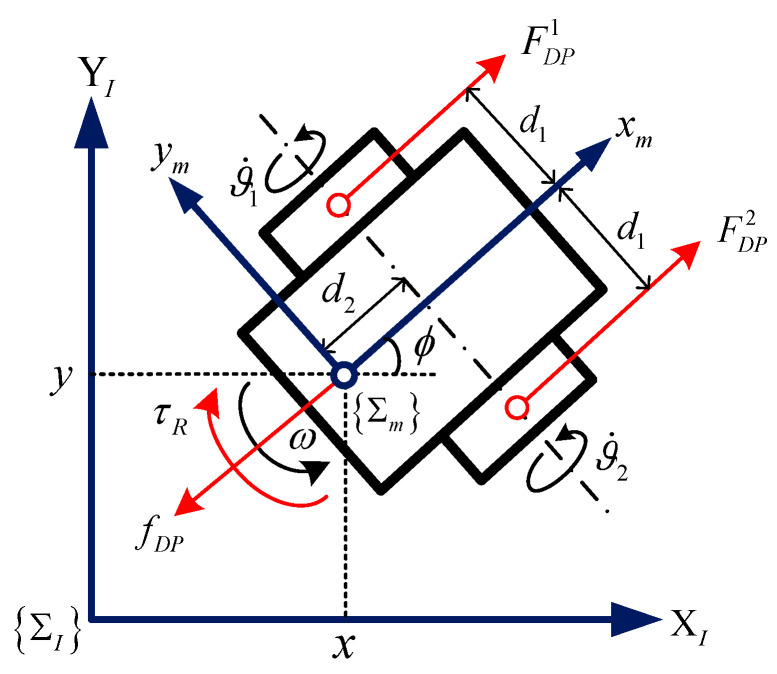
The structure of the wheeled mobile robot.

The WMR system [54] in Figure 2 illustrates the effectiveness of the proposed algorithm.

(102) $$\left\{\begin{array}{l} m\dot{v}\cos\beta_{w}-mv{\dot{\beta }}_{w}\sin\beta_{w}+md_{w}{\dot{\phi }}^{2}=F_{DP}^{1}+F_{DP}^{2}-f_{DP}\\ -mg\sin\theta \cos ο\\ I\dot{\omega }=-F_{DP}^{1}d_{1}+F_{DP}^{2}d_{1}-\tau_{R} \end{array}\right.$$

where m represent the robot mass, and I denotes the rotational inertia around the motion center. $\beta_{w}$ is the angle between the robot speed and the $x_{m}$ axis, and ο is the angle of inclination of the ground environment where the robot is located.

Then, rewrite (102) as a vector form

(103) $$M\dot{v}+Vv+G=B\left(\tau -T_{De}\right)-F_{R}$$

where, according to [55], we have $M=\left[\begin{array}{cc} m\cos\beta_{w} & 0\\ 0 & I \end{array}\right]$, $V=\left[\begin{array}{cc} -m{\dot{\beta }}_{w}\sin\beta_{w} & md_{2}\dot{\phi }\\ 0 & 0 \end{array}\right]$, $v=\left[\begin{array}{l} v\\ \omega \end{array}\right]$, $F_{R}=\left[\begin{array}{c} f_{DP}\\ \tau_{R} \end{array}\right]$, $\tau =\left[\begin{array}{l} \tau_{1}\\ \tau_{2} \end{array}\right]$, $G=\left[\begin{array}{c} mg\sin\theta \cos ο\\ 0 \end{array}\right]$, $B=\frac{1}{r_{s}}\left[\begin{array}{cc} 1 & 1\\ -d_{1} & d_{2} \end{array}\right]$, $T_{De}=\left[\begin{array}{c} \left\{RC_{01}^{s}+k_{RC_{1}}^{s}s_{1}+A_{RC_{1}}^{s}\cos\left(n_{L}\vartheta_{2}-\xi_{01}^{s}\right)\right\}F_{N}^{1}\\ \left\{RC_{02}^{s}+k_{RC_{2}}^{s}s_{2}+A_{RC_{2}}^{s}\cos\left(n_{L}\vartheta_{2}-\xi_{02}^{s}\right)\right\}F_{N}^{2} \end{array}\right]$.

Considering the symmetry of the quality matrix and incorporating the time delay in the state, the state-space form can be used to represent the dynamic system of WMR.

(104) $$\dot{v}\left(t\right)=f\left(t\right)v\left(t\right)+h\left(v\left(t-t_{1}\right)\right)+g\left(t\right)u+\omega \left(t\right)$$

where $f\left(t\right)=-M^{-1}V$, $g\left(t\right)=M^{-1}B$ is an unknown function, and $\omega \left(t\right)=M^{-1}\left(BT_{De}+F_{R}-G\right)$ denotes the resistance torque with the same effect and unknown resistance.

The desired trajectories indicate the forward and steering angular velocity, are given as $x_{d,1}=1.2+0.5\sin\left(0.05t\right)$ and $x_{d,2}=0.5\cos\left(0.05t\right)$.

Depending on the actual WMR system, the initial values are $\beta_{w}\left(0\right)={\left[0,0\right]}^{T}$, $rand\left(1,4\right)$ and $rand\left(1,4\right)$, and $\alpha_{c}=0.13$, $\alpha_{a}=0.12$, λ=0.05, γ=0.10 and R=1, $Q=\left[1,0;0,1\right]$ in this simulation. Then, the following simulation results are presented.

**Figure 3:**
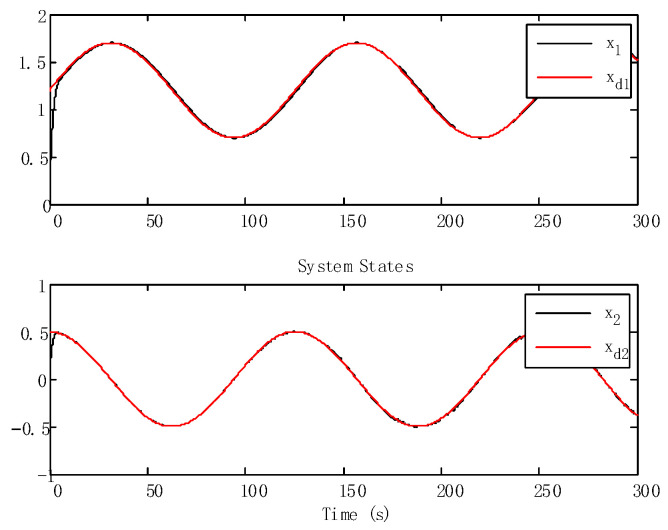
Tracking trajectories of the states.

**Figure 4:**
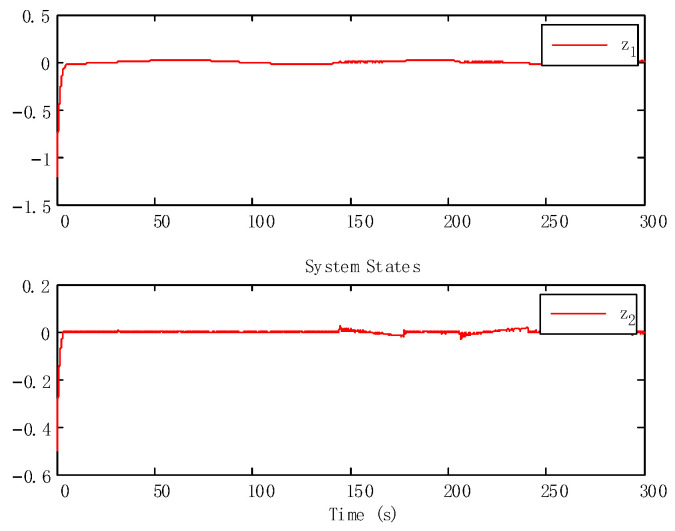
Tracking errors.

With the state time delay handled by appropriate LKFs, the impact of delay is successfully suppressed. From Figure 3 and Figure 4, we can obtain that the tracking performance of the proposed algorithm has good tracking performance.

**Figure 5:**
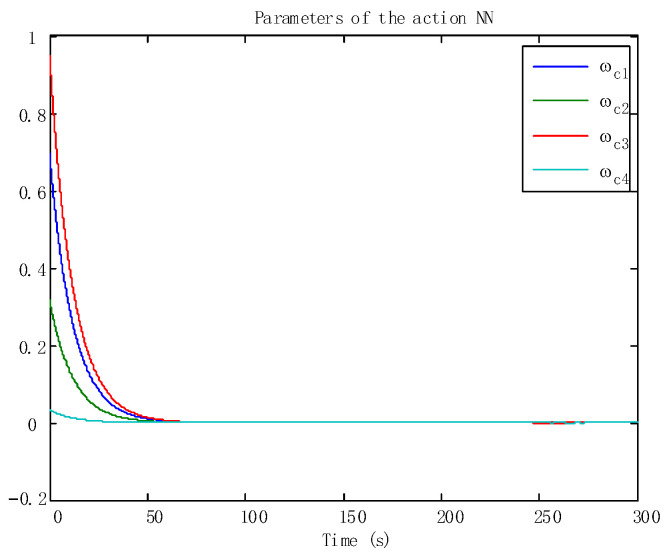
The adaptive laws of the action NNs.

**Figure 6:**
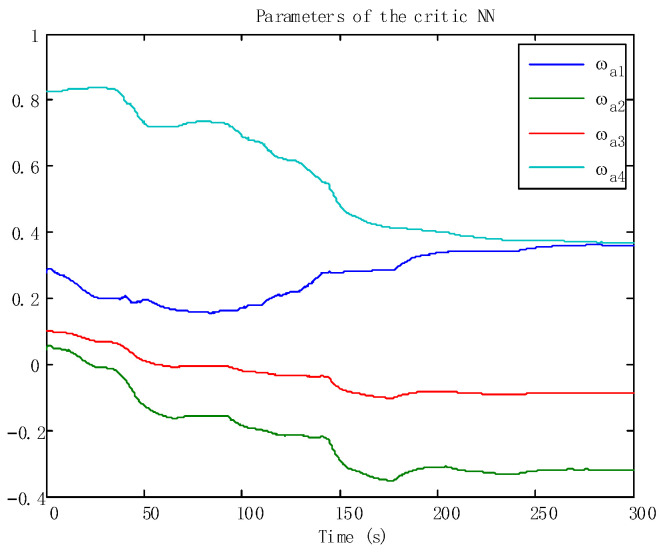
The adaptive laws of the critic NNs.

**Figure 7:**
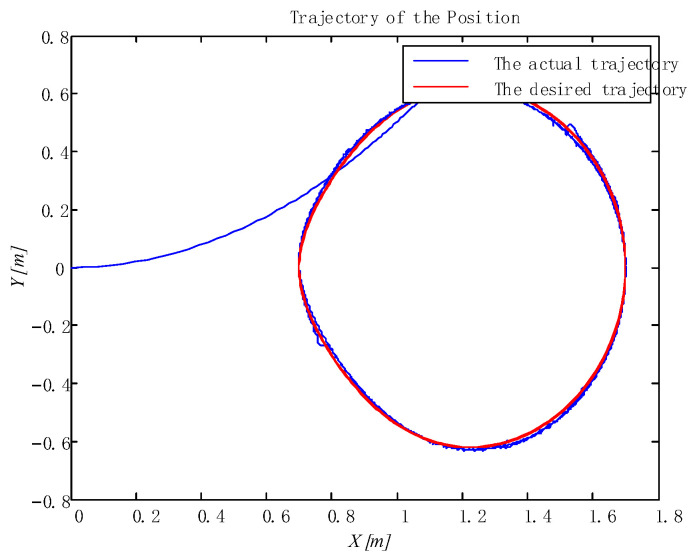
Tracking trajectories of the position.

In addition, the adaptive update of the critic and the action can be reflected in Figure 5 and Figure 6, which ensures the boundedness of the adaptive law. Moreover, the tracking trajectory of the WMR is shown in Figure 7. According to the process, the signal in the wheeled mobile robotic system is SGPFS. Compared with previous work [56], with similar control effects, this paper additionally considers finite-time control and the final simulation results achieve finite-time convergence, reflecting the control advantages of the proposed algorithm.

## 5. Conclusions

A finite-time adaptive online optimization tracking control algorithm was suggested for nonlinear systems incorporating state time delays. By using appropriate LKFs, the issue arising from time delays in both state and input variables has been resolved. Then, a novel nonquadratic HJB function was defined, where finite time was selected as the upper limit of integration, which contains information of the state time delay on the premise of containing the basic information. With the premise of meeting specific requirements, the ideal HJB function was solved by using the IRL method. Furthermore, the SGPFS was guaranteed with the definition of the optimal control policy and the update of the adaptations of the critic and action NNs.

## Data Availability

Data are contained within the article.
